# Translational Diffusion and Self-Association of an Intrinsically Disordered Protein κ-Casein Using NMR with Ultra-High Pulsed-Field Gradient and Time-Resolved FRET

**DOI:** 10.1021/acs.jpcb.4c03625

**Published:** 2024-08-06

**Authors:** Daria L. Melnikova, Venkatesh V. Ranjan, Yuri E. Nesmelov, Vladimir D. Skirda, Irina V. Nesmelova

**Affiliations:** † Department of Physics of Molecular Systems, Kazan Federal University, Kazan 420011, Russia; ‡ Department of Chemistry, University of North Carolina, Charlotte, North Carolina 28223, United States; § Department of Physics and Optical Sciences, University of North Carolina, Charlotte, North Carolina 28223, United States; ∥ School of Data Science, University of North Carolina, Charlotte, North Carolina 28223, United States

## Abstract

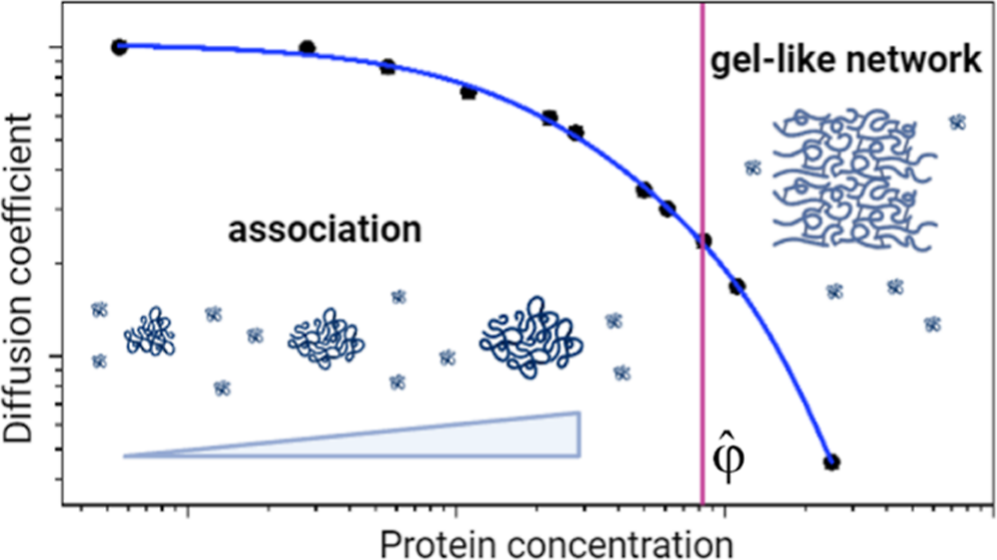

Much attention has been given to studying the translational diffusion of globular proteins, whereas the translational diffusion of intrinsically disordered proteins (IDPs) is less studied. In this study, we investigate the translational diffusion and how it is affected by the self-association of an IDP, κ-casein, using pulsed-field gradient nuclear magnetic resonance and time-resolved Förster resonance energy transfer. Using the analysis of the shape of diffusion attenuation and the concentration dependence of κ-casein diffusion coefficients and intermolecular interactions, we demonstrate that κ-casein exhibits continuous self-association. When the volume fraction of κ-casein is below 0.08, we observe that κ-casein self-association results in a macroscopic phase separation upon storage at 4 °C. At κ-casein volume fractions above 0.08, self-association leads to the formation of labile gel-like networks without subsequent macroscopic phase separation. Unlike α-casein, which shows a strong concentration dependence and extensive gel-like network formation, only one-third of κ-casein molecules participate in the gel network at a time, resulting in a more dynamic and less extensive structure. These findings highlight the unique association properties of κ-casein, contributing to a better understanding of its behavior under various conditions and its potential role in casein micelle formation.

## Introduction

The conformational plasticity of a protein in response to environmental conditions is determined by the physicochemical properties of amino acids and their arrangement in the protein sequence, which dictates which intramolecular interactions or side chain interactions with the solvent are more favorable.^1^ Water is a poor solvent for the protein backbone, and in a natural environment, many proteins adopt a compact three-dimensional structure stabilized by a variety of interactions, such as ionic and hydrophobic interactions as well as hydrogen and disulfide bonds.^2,3^ However, protein sequences with low hydrophobicity and high net charge preferentially adopt disordered, extended conformations.^4−6^ These intrinsically disordered proteins (IDPs) play a functional role in signaling pathways and regulatory processes.^7−9^ Understanding how IDPs move in crowded environments is particularly important because IDPs are commonly found in cellular compartments and regions with high local concentrations of proteins, DNA, and RNA.^10−12^

In the absence of a rigorous theoretical framework, an effective approach to understanding the translational diffusion of IDPs is to compare their translational diffusion coefficients at various concentrations with two well-studied limiting cases: flexible synthetic polymers and globular proteins. In accordance with polymers and globular proteins having fundamentally different structures, the behavior of their diffusion coefficients mirrors these differences, which are particularly evident as solutions transition from dilute to crowded.^13^ The master curve for the concentration dependence of translational diffusion coefficients of synthetic polymers, constructed based on de Gennes’ dynamic scaling theory,^14,15^ is valid for solvent quality ranging from θ (where polymer–polymer interactions equal polymer–solvent and solvent–solvent interactions) to good (where polymer–solvent interactions prevail over polymer–polymer interactions). This master concentration dependence curve shows a gradual increase from dilute solutions, where interactions between molecules are negligible and the polymer molecule moves as an impenetrable to solvent molecules coil, to concentrated solutions, where polymer molecules entangle, and their motion is much more complex. In contrast, the master concentration dependence curve for globular proteins^16^ demonstrates a more sharp transition from dilute to concentrated solutions that follows the theoretical concentration dependence of the diffusion coefficient of rigid Brownian spheres.^17^ Furthermore, the diffusion regime in concentrated solutions of globular proteins qualitatively differs from that of polymers because the maximum solubility of the globular proteins is approximately close to the concentration of close packing for hard spheres, where entanglement is not expected.

Since IDPs do not form well-defined structures, they explore a large number of conformations. An empirical expression *R*_H_ ∼ *N*^ν^, relating the hydrodynamic radius *R*_H_ of an IDP to the number of amino acid residues *N* comprising it, was proposed based on the analysis of experimental data.^18−20^ Reported Flory exponent ν values for IDPs range from 0.49289 to 0.50987 and correspond to the value of ν for homopolymers in θ (indifferent) solvents.^21^ Note that in dilute solutions, even random coils move as hydrodynamically compact species. We have observed such behavior in solutions of the IDP α-casein,^22^ where the diffusion coefficient of α-casein follows the same trend as the concentration dependence of the diffusion coefficients of globular proteins and rigid Brownian spheres. However, increasing IDP concentration can significantly increase their conformational heterogeneity. Additionally, IDPs have much shorter chains and heterogeneous charge distributions along the amino acid sequence, resulting in varying degrees of compaction.^18,23,24^ Therefore, it is unclear whether the diffusion behavior of an IDP in crowded space corresponds to that in a concentrated polymer solution, in which long and fully flexible polymer chains entangle, forming transient networks.

One of the normalization parameters, used in constructing the master curves for synthetic polymers or globular proteins,^16,25^ is critical concentration φ̂. We have shown that in the case of globular proteins φ̂ reflects the tendency of molecules to self-associate.^16^ For example, φ̂ is equal to 0.16 (expressed as a volume fraction) in solutions of myoglobin, where no association is observed, whereas it is equal to 0.08 in the solution of lysozyme at pH 7.4–7.8, where lysozyme molecules form aggregates.^16,26−28^ The shape of the master curve is also sensitive to the key features of the aggregation process. In concentrated solutions, the diffusion coefficient of an IDP α-casein demonstrates a much stronger concentration dependence, φ^–12^, than that of globular proteins or linear flexible polymers.^22^ This strong dependence results from the continuous self-assembly of α-casein molecules into labile supramolecular gel networks, restricting the translational mobility of the molecule as a whole due to the formation of multiple protein–protein interactions that lead to gel formation and are not accounted for in the construction of the master curve.^22^ In this regard, comparing experimental data with master curves allows identifying the presence of interactions leading to the formation of supramolecular structures in the studied solutions. Although not shown before for proteins, based on the data for several polymer systems,^29,30^ we also expect that the concentration dependence of an IDP may be sensitive to liquid–liquid phase separation (LLPS), which is a ubiquitous phenomenon in IDP proteins.^31^ However, due to limited information on the translational diffusion of IDPs in concentrated solutions, both under self-crowding conditions and in the presence of crowding molecules of different nature, more experimental data are needed to assess the impact of various types of self-association on the translational diffusion of IDPs and to determine the applicability of scaling laws and master curve analysis in such conditions.

The goal of this work was to investigate the translational diffusion and supramolecular assembly of κ-casein, an IDP from the casein family.^32−34^ Caseins comprise approximately 80% of milk protein, and their primary function is to serve as a source of amino acids, calcium, and phosphorus.^35^ There are four types of caseins in mammals: α_s1_, α_s2_, β, and κ-casein.^36^ Due to the amphipathic nature of their molecules, containing both polar and hydrophobic domains, all caseins display a strong tendency to self-associate.^37,38^ The degree and type of association vary between different caseins due to differences in amino acid composition and their distribution in the sequence^39,40^ (Supporting Information and Figure S1). κ-Casein is unique among caseins as it has only one or two phosphorylated residues, and thus forms fewer interactions with calcium compared to other caseins and remains soluble in the presence of calcium.^41^ Independently, κ-casein exists as a dynamic, oligomeric ensemble, the properties of which are highly dependent on concentration as well as solution pH and buffer composition.^42−45^ In the mixture, κ-casein interacts with highly phosphorylated α and β-caseins and prevents their aggregation and precipitation in the presence of high concentrations of calcium,^39,46^ leading to the formation of a thermodynamically stable complex with calcium phosphate known as casein micelle.^36,39^ In the casein micelle, κ-casein is believed to play a stabilizing role by forming a polyelectrolyte “brush”, a highly hydrated layer on the surface that provides electrostatic stability to the micelle in good solvents and determines the micelle size by preventing further aggregation of caseins.^47^ Currently, the model describing how a casein micelle forms is still under debate.^41^ Therefore, understanding the association and nature of intermolecular interactions of casein molecules, both with themselves and with each other, remains important.

Pulsed-field gradient nuclear magnetic resonance (PFG NMR) diffusion measurements are particularly suitable for studying protein association,^13,48^ as the translational diffusion coefficient is inversely proportional to the size of diffusing species and, thus, highly sensitive to size changes. However, the informativeness of results on molecular association depends on the ability to measure very slowly moving molecules. Previously, using ultrahigh PFG NMR, enabling us to measure the diffusion coefficients as low as 10^–15^ m^2^/s, we detected and characterized three-dimensional gel-like structures in α-casein solutions.^22,49^ In this work, we utilized ultrahigh PFG NMR to investigate κ-casein solutions and found that the molecules of κ-casein also form geometrically similar gel-like networks in concentrated solutions but exhibit fundamentally different behavior from α-casein at concentrations below the gel formation threshold. Specifically, we observed self-association of κ-casein molecules even at very low protein concentrations and macroscopic phase separation when solutions were stored.

## Methods

### Materials

Bovine κ-casein (C0406) was purchased from Sigma-Aldrich and used without further purification. We verified the purity and homogeneity of κ-casein by gel electrophoresis [sodium dodecyl sulfate–polyacrylamide gel electrophoresis (SDS–PAGE)]. Under reducing conditions, κ-casein migrated as a single band at 19 kDa (Figure S2), which corresponds to its molecular weight. We also confirmed that κ-casein is unstructured by circular dichroism spectroscopy (Figure S3). All measurements were carried out using fresh samples within several hours after preparation, except otherwise mentioned.

### PFG NMR Diffusion Measurements

For NMR diffusion measurements, the lyophilized powder of κ-casein was dissolved in D_2_O to minimize the signal from water protons in NMR spectra. Protein concentrations ranged from 0.1 to 20% (w/v %, hereafter) or 0.001 to 0.147 volume fractions. The volume fraction, φ, of κ-casein was calculated using the following relation
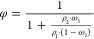
1where ρ_1_ and ρ_2_ are the densities of water and κ-casein, respectively, and ω_1_ is the weight fraction of water. The density of κ-casein was calculated using its partial specific volume value of 0.689 cm^3^/g, determined previously.^50,51^

All NMR measurements were performed at 298 K on a 400 MHz Bruker AVANCE-III TM spectrometer equipped with a gradient system that allowed an ultrahigh gradient, *g*, with the maximum value of 28 T/m (2800 G/cm). Self-diffusion coefficients (hereinafter referred to as diffusion coefficients) were measured using the stimulated-echo pulse sequence (STE)^52^ and the modified five-pulse stimulated echo sequence (MODSTE)^53^ (Figure S4). The integrated area of the protein peak between 0.16 and 3.61 ppm was used to characterize the κ-casein signal. The experiments were carried out using 48 different values of *g* and gradient pulse durations δ of 1, 2, or 5 ms. The time interval between the first and second radiofrequency (RF) pulses was kept constant in all experiments at τ_1_ = 10 or 16 ms to exclude the influence of spin–spin relaxation time *T*2 on the shape of diffusion attenuation. The diffusion time *t*_d_ varied from 50 to 800 ms by changing τ_2_ in the MODSTE pulse sequence (Figure S4). The standard experimental error of measured diffusion coefficients was below 5%.

Multiexponential diffusion attenuations were described by the spectrum of diffusion coefficients, *D*_i_, according to the equation
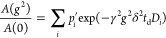
2where *A*(0) is the spin–echo amplitude at *g* = 0, γ is the gyromagnetic ratio for protons, and *p*_*i*_^′^ is the relaxation-weighted fraction of the component with the diffusion coefficient *D*_i_ given by the equation
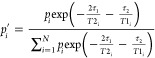
3where *p*_*i*_ is the fraction of the component with the diffusion coefficient *D*_*i*_, *T*2_*i*_ and *T*1_*i*_ are the proton spin–spin and spin–lattice relaxation times of κ-casein molecules within an *i*-th aggregate, and τ_2_ is the interval between the second and third 90° RF pulses in a stimulated-echo pulse sequence.

To describe the spectrum of observed diffusion coefficients, we used the average diffusion coefficient ⟨*D*⟩ defined in accordance with the equation
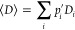
4⟨*D*⟩ is determined with high accuracy from the initial slope of the diffusion attenuation (*g* → 0).

In the presence of molecular exchange between species with different diffusion coefficients, to evaluate the fraction of molecules and their lifetime within a given species, it is necessary to maintain the contribution of relaxation factors (eq 3) constant for different diffusion times. This was achieved using the MODSTE pulse sequence presented in Figure S4. In this pulse sequence, by maintaining the τ_2_ + τ_4_ sum constant, the diffusion attenuation can be recorded at different diffusion times while keeping the contribution of *T*1 the same. We demonstrated that the MODSTE pulse sequence enables the unambiguous characterization of the exchange process between species with different diffusion coefficients *D*_*i*_, even if each of the exchanging species is also characterized by distributions of both T1 and T2 relaxation times, by applying this approach to evaluate the lifetime of a “guest-host” complex formed by the antitumor agent 5-FU and carrier β-CD.^53^

To obtain the diffusion coefficient distribution from nonexponential diffusion attenuations, we used the estimate of lifetime and a home-written software based on the Tikhonov regularization algorithm.^54,55^ The main advantage of this software is the minimal number of fitting parameters. Initially, from the analysis of the diffusion attenuation, a physically justified range of expected values for diffusion coefficients is set. The primary regularization parameter, which determines the accuracy of the fit of the diffusion attenuation with the calculated distribution of diffusion coefficients, is the number of iterations *N*_*i*_. The effect of *N*_*i*_ on the spectrum for both nonexponential (0.1% aqueous solution of k-casein) and exponential (water) diffusion attenuations is demonstrated in Figure S5. In our fits, *N*_*i*_ was set to 100.

### Time-Resolved FRET Measurements

For Förster resonance energy transfer (FRET) measurements, the lyophilized powder of κ-casein was dissolved in H_2_O and incubated with the donor (EDANS-C2-maleimide, Anaspec) or acceptor (DABCYL-C2-maleimide, Anaspec) at room temperature for 2 h. The labeling was done at a 1:1 protein/label ratio, and unreacted labels were removed using Amicon ultra centrifugal filters. We assume that only one cysteine per protein was labeled. For titration experiments, the concentration of the donor-labeled κ-casein was kept constant at 0.01% (5 μM), and the concentration of the acceptor-labeled κ-casein varied from 0.01 to 0.38% (5–200 μM).

Time-resolved FRET was measured using a home-built transient fluorimeter equipped with a QuadraCentric sample compartment with a cuvette holder and a Peltier element for temperature control (Horiba Scientific), a passively *Q*-switched microchip YAG laser (SNV-20F-100, 355 nm, 20 kHz, Teem Photonics), a photomultiplier (H6779-20, Hamamatsu), and a fast digitizer (Acqiris DC252, Agilent). A 420 nm cutoff filter and a polarizer set at the magic angle were used in the detection arm. All experiments were done at the temperature of 293 K. The labeled protein solution was loaded into the observation cuvette, and the time-resolved donor fluorescence waveform was acquired by averaging fluorescence transients from 1000 laser pulses. We used a nonfluorescent acceptor to analyze the donor fluorescence only. The obtained waveforms of donor fluorescence were best fitted by three exponential components, convoluted with the instrument response function measured separately from the light scatter before each experiment. The component with the shortest fluorescence decay time (τ_D_ = 0.7 ns) remained constant during the titration at all concentrations of acceptor-labeled protein. The other two components showed an identical decrease of τ_D_. Because the component with the longest decay time comprised approximately 70% of the measured waveform, it was used to extract the values of the donor fluorescence decay time for further analysis. FRET efficiency was determined from each independent experiment using the equation
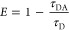
5where τ_D_ is the fluorescence decay time of the donor-labeled protein alone, and τ_DA_ is the fluorescence decay time of the donor-labeled protein in the presence of bound acceptor-labeled protein. The Förster distance, *R*_0_, in our experiments was calculated to be 2.6 nm (Supporting Information).

To describe κ-casein self-association, we used the model of indefinite isodesmic association where the addition of each successive monomer to an associate involves an equal change in free energy. In this model, the following set of equilibria is considered
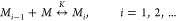
6where *M* denotes a monomer, and *M*_*i*_ denotes an *i*-mer. The total molar concentration of protein, *C*, is the sum of the molar concentrations of all *i*-mers in solution, which can be written using the molar concentration of monomers under the assumption of isodesmic association
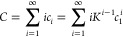
7

For indefinite association, the summation of series gives a simple expression for the total concentration
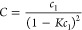
8

From the quadratic equation, the concentration of monomers as a function of total protein concentration is then found
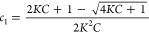
9

Using eq 8, molar fractions for each species in solution can be found according to the following relations
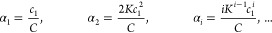
10

The FRET efficiency of the *i*-th κ-casein species in the presence of multiple acceptors is given by the equation^56^
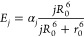
11where *R*_0_ is 2.6 nm. Index *j* indicates the number of acceptors near a donor, and *j* = 1 corresponds to one acceptor near a donor. Accordingly, the cumulative FRET efficiency detected experimentally is written as a sum of all individual contributions
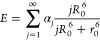
12

## Results

**Figure 1:**
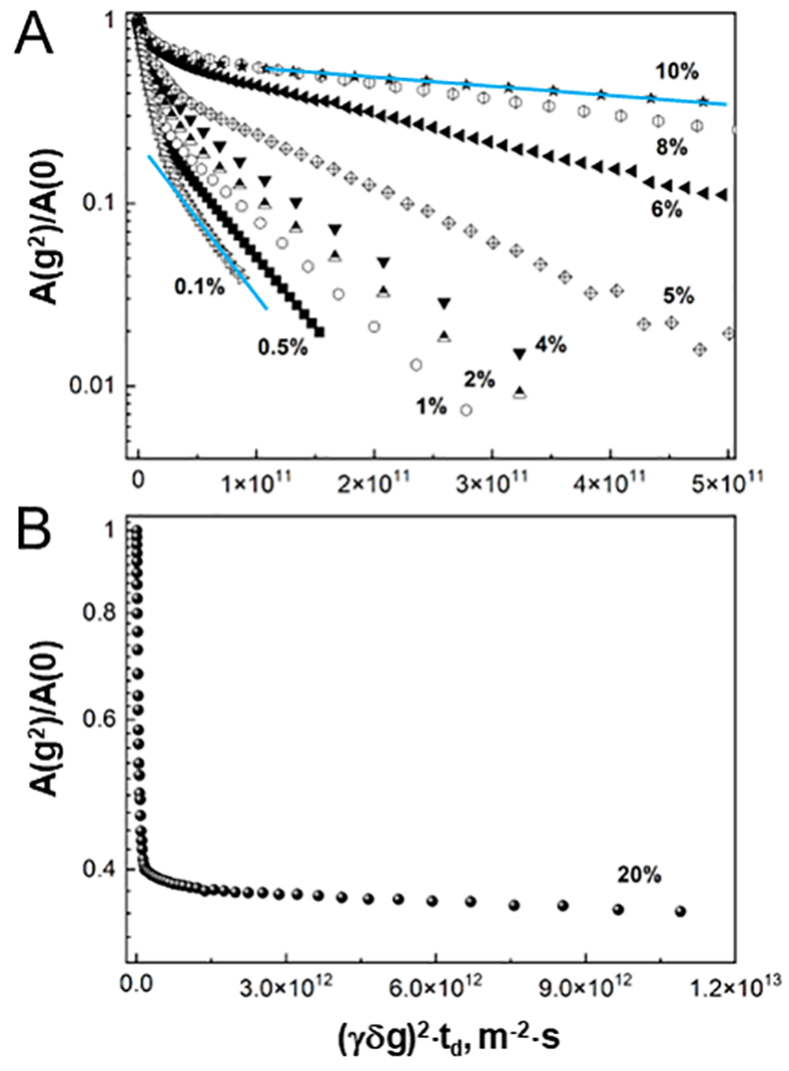
Diffusion attenuations of spin–echo signal in solutions of κ-casein. Diffusion attenuations, recorded at *t*_d_ = 50 ms, are shown for protein concentrations in the range from 0.1 to 10% (A) and 20% (B). Solutions were prepared in 100% D_2_O at pH 7.0. The measurements were done at 298 K. The deviation from a monoexponential attenuation is observed for all protein concentrations. *D*_min_ decreases as the concentration of κ-casein indicated by solid blue lines drawn to 0.1 and 10% curves.

Figure 1 presents the semilogarithmic plots of spin–echo intensity recorded as a function of the pulsed field gradient amplitude, g, at κ-casein concentrations ranging from 0.1 to 20%. The chosen semilogarithmic coordinates clearly reveal the deviation of the signal attenuation curves, *A*(*g*^2^), from monoexponential behavior at all concentrations studied. Since the κ-casein samples are monodisperse in molecular weight (Figure S2), the nonexponential nature of the diffusion attenuation reflects the high propensity of κ-casein to self-associate. As expected for self-associating molecules, as the protein concentration in solution increases, the change of the shape of diffusion attenuations reflects the emergence of increasingly smaller diffusion coefficients. This is clearly observed by the change in the slope of the corresponding part of the diffusion attenuation, as exemplified by blue lines for the 0.1 and 10% solutions.

To verify that the observed self-association in our experiments is not due to the formation of disulfide bonds, previously suggested as one of the mechanisms of casein micelle formation,^57^ we performed the reversibility test as described before.^22^ Since the formation of disulfide bonds is irreversible and is expected to be more pronounced at higher protein concentrations, we compared the diffusion attenuations of 5% κ-casein samples prepared freshly and by dissolving a 20% sample. Both diffusion attenuations were identical, ruling out the formation of associates due to intermolecular disulfide links (Figure S6).

**Figure 2:**
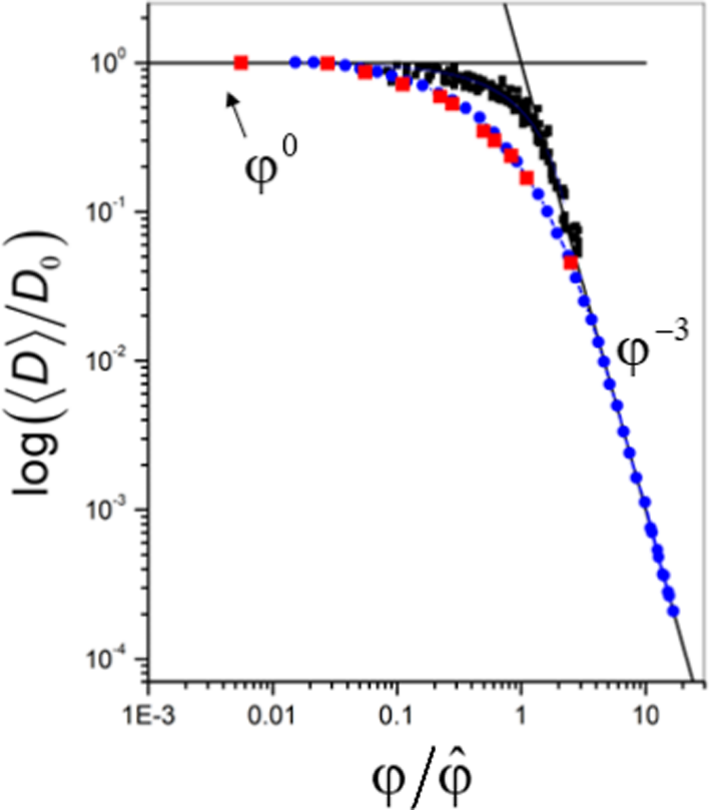
The concentration dependence of the κ-casein diffusion coefficient. The normalized concentration dependence of the normalized κ-casein diffusion coefficient ⟨*D*⟩ is shown by red squares. For comparison, the master curves are shown for the concentration dependence of the diffusion coefficient of linear flexible polymers (blue circles) and globular proteins (black squares). Solid lines indicate the asymptotes with the slopes of φ^0^ and φ^–3^.

To compare the concentration dependence of the translational diffusion coefficient of κ-casein to flexible polymers and globular proteins, we used the average diffusion coefficient ⟨*D*⟩ (eq 4), determined from the initial slope of the diffusion attenuation. The value of ⟨*D*⟩ is several orders of magnitude larger than *D*_min_ and, therefore, primarily reflects the contribution of fast moving κ-casein molecules. Furthermore, without using ultrahigh pulsed-field gradients, all available information on self-diffusion of κ-casein would rely only on average diffusion coefficients. Figure 2 shows the dependence of ⟨*D*⟩ presented in logarithmic coordinates on protein concentration, recalculated as volume fraction using eq 1 to facilitate the comparison with master curves for globular proteins and flexible polymers. We also performed the same normalization procedure established for flexible synthetic polymers and later applied to globular proteins.^15^ First, to eliminate temperature dependence, we divided the diffusion coefficient of κ-casein at each concentration by the value of the diffusion coefficient at infinite dilution, determined by extrapolating the experimental data to zero κ-casein concentration. Then, the volume fraction was normalized by the critical concentration, determined from the intersection of the asymptotes with zero slope (dilute solutions) and slope φ^–3^ (concentrated solutions), as indicated by the solid lines. The value of φ̂ was equal to 0.08. In agreement with observed self-association of κ-casein, this value was smaller than 0.16, determined previously for solutions of nonassociating globular proteins.^16^ The φ^–3^ asymptote was chosen because this asymptotic behavior was theoretically predicted for the diffusion coefficient of synthetic polymers and empirically determined for globular proteins. The normalization to critical concentration is equivalent to shifting the entire curve along the log(φ) axis without changing its shape. Similarly to globular proteins and α-casein,^16,22^ normalization of the diffusion coefficient by the contribution of internal dynamics, as done for flexible polymers, was not necessary.

Remarkably, the concentration dependence of the κ-casein diffusion coefficient shows the same gradual increase as the master curve for flexible synthetic polymers over the entire range of concentrations studied.

Our data, indicating that the κ-casein self-associate even at the lowest used concentration of 0.1%, agree with gel-filtration data^58^ and with reports that reduced and carboxymethylated bovine κ-casein self-associates into micelle-like structures at concentrations above 0.05%.^42,59^ In contrast, no association of α-casein was observed at protein concentrations up to 2%, and the diffusion attenuations for these α-casein solutions were monoexponential.^22^

The value of *D*_min_ in a 0.1% κ-casein solution is approximately 1.5 ± 0.06 × 10^–11^ m^2^/s, which is about an order of magnitude lower than the expected diffusion coefficient for κ-casein monomers, based on comparisons with proteins of similar size.^13,16^ Using the Stokes–Einstein formula, *D* = *kT*/6 πη R, where *k* is the Boltzmann constant, *T* is the temperature, and η is the viscosity of the pure solvent (D_2_O, 1.1 × 10^–3^ Pa·s^60^), we roughly estimate that the hydrodynamic radius *R* of species diffusing with the diffusion coefficient *D*_min_ is approximately 13 nm. This is more than three times greater than the expected value of κ-casein *R*_H_, which is 3.56 nm, estimated using the empirical expression^20^*R*_H_ = 2.84*N*^0.493^ with *N* = 169. We also obtained similar linear dimensions for a κ-casein molecule using a secondary structure prediction method PSIPRED^61^ (Figure S7). Consequently, considering a simplified geometrical model of random close packing of spheres to account for void volume,^62^ the number of κ-casein molecules in the associated species can reach up to about 31. This is in agreement with the polymerization value of 30 determined for κ-casein from viscosity and sedimentation data^59^ and within the range of sizes reported for κ-casein associates measured by different experimental techniques.^36,43,63^

The diffusion attenuation of residual H_2_O in a 0.1% κ-casein solution is monoexponential and does not show *t*_d_ dependence. This could indicate that either the water content in the κ-casein associate is small (below the detection sensitivity) and/or the exchange with bulk water is fast on the time scale of our experiments (≪50 ms). Additionally, this suggests that κ-casein associates are protein-dense structures without a large volume of confined water, which would be expected to demonstrate the features of restricted diffusion.

**Figure 3:**
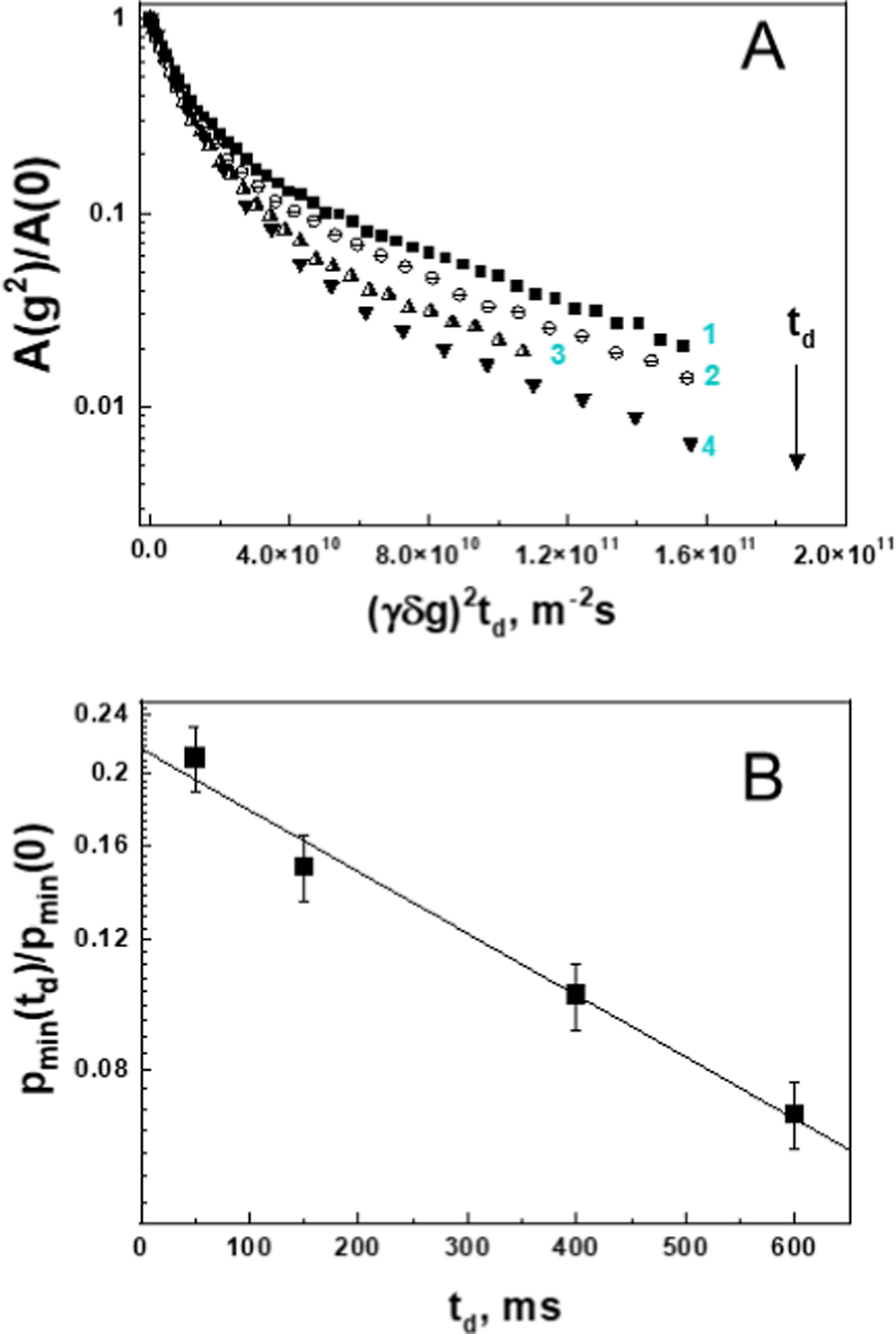
The dependence of the diffusion attenuation on diffusion time in 0.5% κ-casein solution. (A) Curves 1–4 represent diffusion attenuations collected at *t*_d_ = 50, 150, 400, and 600 ms, respectively. (B) The dependence of the fraction of slowly diffusing κ-casein species, *p*_min_, on *t*_d_. The solid line shows the best fit of experimental data to eq 13.

To explore the lability of associated κ-casein species, we studied the dependence of diffusion attenuation *A*(*g*^2^) on diffusion time *t*_d_ for a 0.5% κ-casein solution and assessed the fraction and lifetime of κ-casein molecules within the associates. To exclude the influence of spin–lattice relaxation time *T*1 on the shape of diffusion attenuation (eq 3), we used the modified five-pulse stimulated echo pulse sequence MODSTE.^53^Figure 3A shows diffusion attenuations recorded at different *t*_d_ values. The values of the average and the lowest detected diffusion coefficients ⟨*D*⟩ and *D*_min_ do not depend on the diffusion time. In contrast, the fraction of the slowest diffusing molecules, *p*_min_, decreases with increasing diffusion time, indicating the presence of molecular exchange between different κ-casein species. We estimated the average lifetime τ* of κ-casein molecules in the associated state using the following approach. The probability for a κ-casein molecule to leave the associate species diffusing with *D*_min_ at least once is given by the integral , where τ is the lifetime distribution of κ-casein molecules for an *i*-th snapshot of the system. In this case, the dependence of *p*_min_ on *t*_d_ can be calculated according to the equation
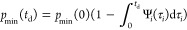
13where *p*_min_(0) is the fraction of the κ-casein molecules in the associates at *t*_d_ approaching zero. Figure 3B shows that the dependence *p*_min_(*t*_d_) can be well described by an exponential function. By fitting the *p*_min_(*t*_d_) data with an exponent, we determined the fraction and lifetime of κ-casein molecules within the associate to be equal to *p*_min_(0) = 0.230 ± 0.002 and τ* = 0.53 ± 0.06 s, respectively. Note that the value of *p*_min_(0) includes the relaxation contribution as described by eq 3, and thus provides the lower limit estimate.

**Figure 4:**
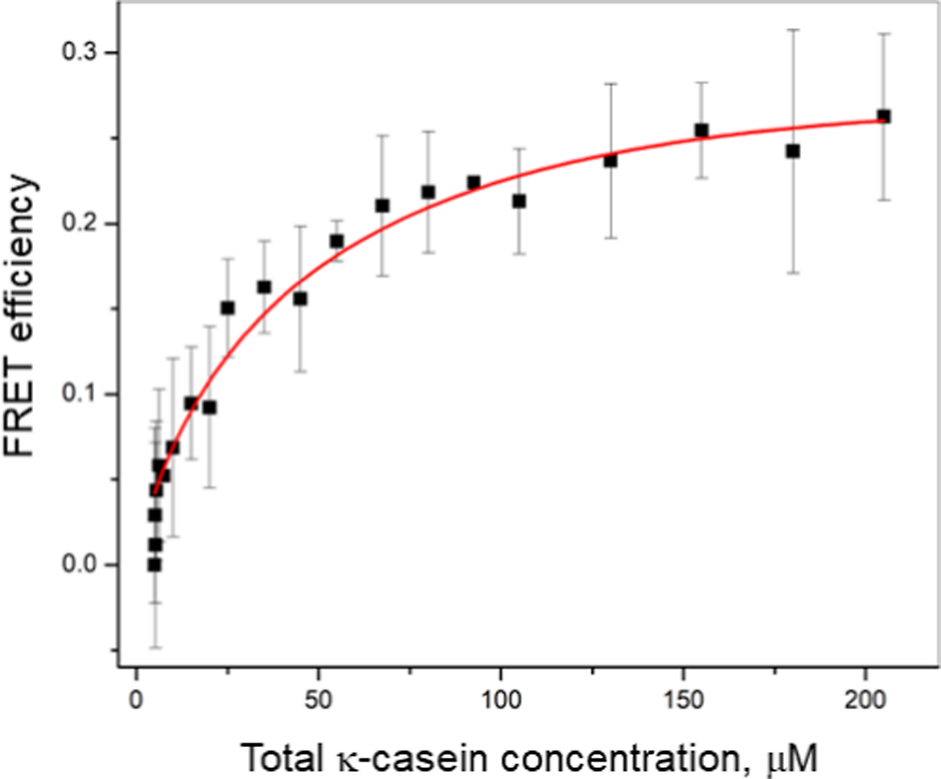
The dependence of FRET efficiency on κ-casein concentration. Experimental error is shown as standard deviation from at least three independent measurements. The solid line represents the best fit of the isodesmic association model to experimental data.

To corroborate the NMR diffusion data and determine the dissociation constant for κ-casein species forming in dilute solutions, we carried out FRET titration experiments. During the titration, the concentration of the donor-labeled κ-casein (EDANS-labeled) was kept constant at 0.01% (5 μM), whereas the concentration of the acceptor-labeled κ-casein (DABCYL-labeled) was incrementally increased from 0.01 to 0.38% (5–200 μM). Figure 4 shows that FRET efficiency increases as a function of κ-casein concentration, reflecting the formation of donor–acceptor complexes.

Using FRET efficiency data, we estimated the dissociation constant for adding a monomer to an associate of κ-casein under the assumptions of indefinite isodesmic association and the contribution of acceptors in the first layer around the donor only. The latter assumption is based on the fact that the linear dimensions of the κ-casein molecule are comparable to *R*_0_ = 2.6 nm (see Supporting Information), and the FRET efficiency for the donor–acceptor pair at a distance of ∼2*R*_0_ apart becomes less than 2%. By fitting eq 12 to the experimental data (solid red line in Figure 4), the value of the κ-casein equilibrium dissociation constant, *K*_D_ (the inverse of K, eq 7), is estimated to be 9.5 ± 1.5 μM. We note that the self-association of κ-casein is highly sensitive to buffer conditions. We provide the estimate for the aqueous solution of κ-casein, whereas the addition of 5 mM sodium phosphate buffer leads to about a 3-fold decrease in the equilibrium association constant (e.g., stronger affinity of binding, data not shown). Given the difference in experimental conditions, our *K*_D_ value reasonably agrees with surface plasmon resonance data for casein–casein interactions.^58^

**Figure 5:**
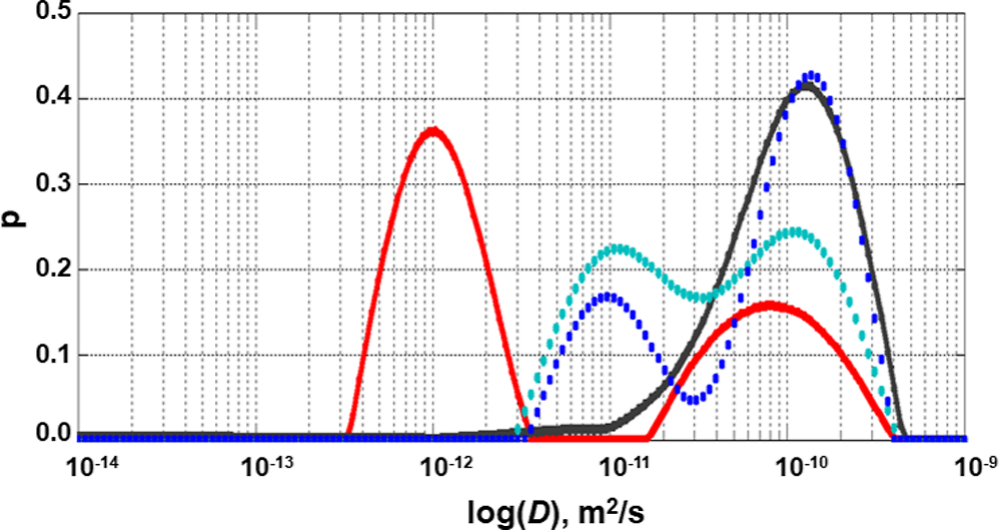
The distribution of diffusion coefficients in κ-casein solutions. The diffusion coefficient spectra are shown for 0.1% (black), 1% (blue), 4% (cyan), and 10% (red).

Using the estimate of a lifetime and a home-written software based on the Tikhonov regularization algorithm,^54^ we calculated the spectra of diffusion coefficients from the nonexponential diffusion attenuations, recorded for κ-casein solutions in the range of concentrations from 0.1 to 10% (Figure 5). These spectra clearly show a bimodal distribution of molecules into slowly and fast-diffusing, with the fraction of slowly diffusing molecules increasing with the increasing concentration of κ-casein.

**Figure 6:**
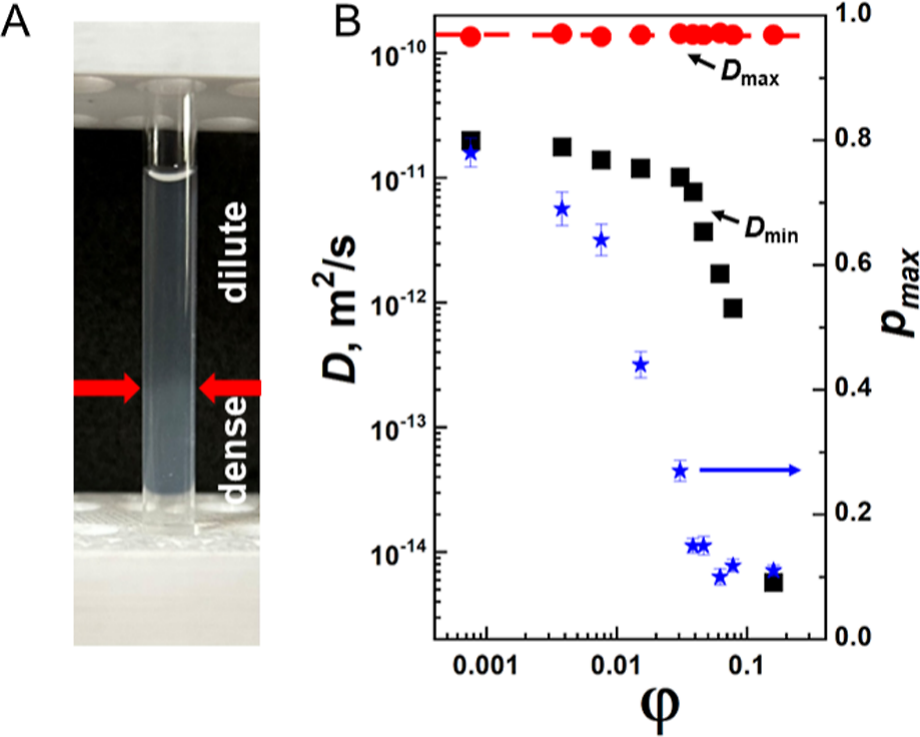
Phase separation in κ-casein solution. (A) Image of κ-casein sample, taken at ambient room temperature immediately after removal from 4 °C, shows the separation in dilute and condensed phases. (B) The dependence of *D*_max_ (red symbols) and *D*_min_ (black symbols) (left vertical axis) and *p*_max_ (blue symbols, right vertical axis) on κ-casein volume fraction φ.

We noticed that incubation of κ-casein solutions with protein concentration below 10%, but not above 10%, at 4 °C for more than 6 days led to visible changes characteristic of phase separation in the sample. Solutions, which were initially transparent, turned cloudy, with two layers becoming visible (Figure 6A). At the same time, the spin–spin relaxation exhibited two relaxation times for the protein, with the shortest time corresponding to species with the slowest diffusion coefficient, characterizing the dense phase.

Since high gradients allowed us to register diffusion coefficients in both the dilute (*D*_max_) and dense phases (*D*_min_), we tracked their changes depending on the sample concentration. Figure 6B shows that the diffusion coefficient of κ-casein in the dilute phase remains unchanged within the limits of experimental error over the entire concentration range. This indicates that as the concentration of κ-casein increases, κ-casein molecules enter the concentrated phase while the concentration of phase depleted of κ-casein remains constant. At the same time, the relative population of the dilute phase decreases as indicated by the decrease of the population *p*_max_ of the component in the diffusion attenuation characterized by *D*_max_. The minimum diffusion coefficient of the condensed phase decreases with increasing protein concentration, as expected, considering typical effects of concentration, such as the increase in solution viscosity. A clear transition point on the concentration dependence of *D*_min_ is observed, indicating the possibility of a qualitatively different diffusion regime.

**Figure 7:**
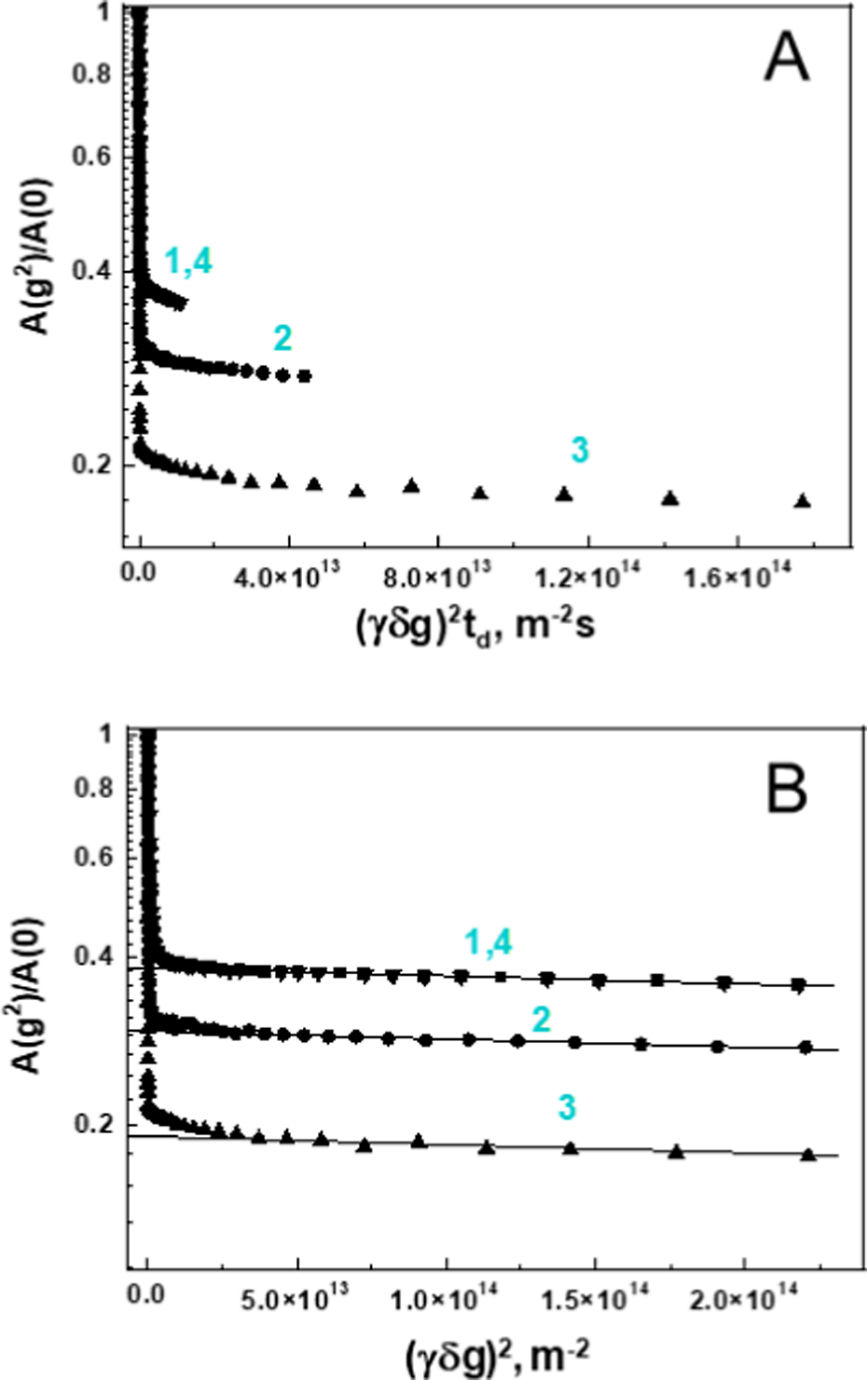
Diffusion attenuation for 20% κ-casein solution. (A) Curves 1–3 represent diffusion attenuations collected at *t*_d_ = 50, 200, and 800 ms, respectively. Curve 4 is a control. It was collected at *t*_d_ = 50 ms after the completion of experiments carried out at different values of *t*_d_ and coincides with curve 1, indicating no changes to the sample during the measurement time. (B) Curves 1–4 from panel A are replotted using coordinates log(*A*(*g*^2^)/*A*(0)) vs (γδ*g*)^2^ to evaluate the dependence of the diffusion coefficient on diffusion time.

We next investigated the translational diffusion in the 20% κ-casein solution. Figure 7A shows multiexponential diffusion attenuations of the spin–echo signal recorded at different diffusion times for a 20% κ-casein solution using MODSTE pulse sequence. The initial slope of the diffusion attenuation remains unchanged, while the fraction of the slowest diffusing molecules, *p*_min_, decreases with increasing diffusion time. As with the 0.5% κ-casein solution, the decrease of *p*_min_ with increasing diffusion time in the 20% κ-casein solution is caused by molecular exchange. However, in contrast to the 0.5% κ-casein solution, we observed the change of the diffusion coefficient *D*_min_ with diffusion time. The dependence of *D*_min_ on *t*_d_ is readily revealed by replotting diffusion attenuations using coordinates log[A(g^2^)/A(0)] vs *kt*_d_ (Figure 7B). In these coordinates, the slope of the slowest-diffusing component remains constant at all values of *t*_d_, indicating that *D*_min_ is inversely proportional to the diffusion time. Accordingly, based on the Einstein relationship between the diffusion coefficient and the root-mean-square (RMS) displacement, ⟨*r*^2^⟩ = 6*t*_d_·*D*_min_, the RMS displacement of κ-casein molecules remains constant, indicating that in the investigated *t*_d_ range in a 20% solution the molecules of κ-casein undergo anomalous, fully restricted diffusion. The estimated size of the restrictions is ≈42 ± 4 nm, which is about an order of magnitude greater than the hydrodynamic radius of κ-casein (3.56 nm). This value is comparable to the size of restrictions in the gel-like network of α-casein (50 ± 5 nm), indicating a geometrical similarity between their structures.

The observation of fully restricted diffusion, in the range of *t*_d_ from 50 to 800 ms, in the 20% solution of κ-casein suggests the formation of a three-dimensional gel network, in which individual κ-casein associates, already present in dilute solutions, interact with each other, losing their mobility as a whole.^22,25,29,30^ In this scenario, the diffusion coefficient *D*_min_ is associated with the movement of segments between the points of contact in the gel network. Using the dependence of *p*_min_ on *t*_d_, we estimated that 34% of κ-casein molecules join the gel network, and their lifetime within the network is equal to 0.98 ± 0.08 s. This value is about twice as large as the lifetime of κ-casein molecules within associates in a 0.5% κ-casein solution. In the case of α-casein, ∼93% of its molecules joined gel-like network and the lifetime in the associated gel state was about 3.5 s. Accordingly, the gel-like network formed by κ-casein is less extensive and more dynamic than that of α-casein.

Additionally, the state of 10–20% samples does not visibly change on storage time as the samples remain transparent and do not show any indication of macroscopic phase separation. Apparently, the formation of gel network restricts the κ-casein capability to phase separate, e.g. by imposing spatial restrictions.

## Discussion and Conclusions

In this study, we examined the self-association behavior and translational diffusion of κ-casein in aqueous solutions across a broad concentration range, from 0.1 to 20%. Collectively, our data show that κ-casein self-associates over the entire concentration range. The associated structures are labile, as indicated by the exchange between κ-casein molecules in the associated state and the bulk solution. In the 20% κ-casein solution, these associated species further aggregate, forming a three-dimensional gel network, where κ-casein molecules remain about twice as long as in the associated states in solutions without gel formation. Additionally, in solution with concentrations below threshold of gel formation, the translational diffusion of κ-casein is unrestricted, whereas in the 20% solution, while the molecule remains in the gel network, it is fully restricted.

Only about a third of κ-casein molecules join the gel network at a time, which is the average dynamic equilibrium number of κ-casein molecules in the gel state. This allows us to compare the translational diffusion of remaining free κ-casein molecules, characterized by ⟨*D*⟩, to that of the closely related IDP α-casein^22^ and to master curves for globular proteins^16^ and flexible polymers^15^ across different concentrations. The concentration dependence of the κ-casein diffusion coefficient differs from that of α-casein or globular proteins but follows the master curve for flexible polymers (Figure 2). The main difference between the master curves for flexible polymers and globular proteins is that for polymers, the transition from dilute to concentrated solutions is smoother and more gradual. The concentration dependence of the κ-casein diffusion coefficient demonstrates the same gradual transition. We attribute this behavior to the continuous self-association of κ-casein observed across the entire concentration range. Assuming that at larger concentrations, the associates of larger size are increasingly frequent, the average diffusion coefficient reflecting the whole distribution of diffusion coefficients (eq 4) would gradually decrease. To explain the alignment of the κ-casein ⟨*D*(φ)⟩ dependence with the master curve of flexible polymers, we speculate that κ-casein can be equivalent to polymers that exist as heterogeneous species characterized by molecular weight (and size) distribution. Thus, the shape of the ⟨*D*(φ)⟩ dependence is sensitive to mass heterogeneity and molecular self-association. In contrast, globular proteins or α-casein do not show self-association in dilute solutions, with the onset of self-association being more discernible. Interestingly, the master curve for three high-generation poly(allylcarbosilane) dendrimers^64^ follows the master curve for globular proteins over the entire concentration range evaluated, suggesting that the sharp transition from dilute to concentrated solutions is characteristic of monodisperse hydrodynamically compact molecules. Note that although the ⟨*D*(φ)⟩ dependence does not show a sharp crossover due to the effect of self-association, our data do not fully rule out the relative compactness of κ-casein molecules, even though they form many intermolecular contacts and join associates. Overall, we note, however, that applying the scaling law without a detailed investigation of the diffusion attenuation shape using ultrahigh pulsed-field gradients would not enable discerning specific details of the translational diffusion in κ-casein solutions, such as gel formation or phase separation.

The behavior of the κ-casein diffusion coefficient ⟨*D*⟩ differs from that of α-casein in concentrated solutions.^22^ The concentration dependence of the α-casein diffusion coefficient has an asymptotic behavior of φ^–12^. The reason for such a strong concentration dependence of the α-casein diffusion coefficient is the formation of a three-dimensional gel network, supported by a fine balance of electrostatic repulsion and attractive hydrophobic interactions. Previously, a deviation of the concentration dependence of the diffusion coefficient from the master curve due to gel formation was also observed for several polymer systems, including gelatin-water and cellulose triacetate-benzyl alcohol.^25,29,30^ Unlike α-casein, the diffusion coefficient of κ-casein does not show such a strong dependence on concentration, despite the formation of a geometrically similar gel structure with comparable restriction sizes of about 50 nm. Besides the effect of continuous self-association, other factors partially explaining the observed difference may include the smaller number of κ-casein molecules joining the gel network (∼34 vs ∼93% in the case of α-casein) and faster molecular exchange (the lifetime of α-casein in the bound state is 3.5 s). The translational diffusion coefficient directly reflects the size of the diffusing species, and our study clearly demonstrates that it allows insight into their self-association processes. As the experimental data on concentration dependence of different IDPs accumulate, it will become clear whether a general scaling law can be established for IDPs as it has been done for synthetic polymers, dendrimers, or globular proteins.

Considering the nonexponential shape of the spin–echo diffusion attenuation, we observe the pronounced separation of κ-casein molecules into fast and slow in terms of their translational mobility that progressively increases with time in solutions with κ-casein concentration below 10%. Macroscopic phases become visually apparent upon the incubation of κ-casein samples at 4 °C for more than 6 days more than 6 days, with a clear boundary between the dilute and dense phases (Figure 6A). In this regard, the association observed in freshly made samples could be interpreted as the formation of microscopic LLPS before layer separation. The dense phase is characterized by extensive intermolecular contacts,^65^ significantly slowing down molecular translational diffusion. Previous PFG NMR studies demonstrated up to 2 orders of magnitude reduction in translational diffusion coefficient of a 103-residue disordered region of CAPRIN1 protein^66^ or intrinsically disordered N-terminal 236 residues of the germ-granule protein Ddx4.^67^ Our data show a similar level of reduction in translational diffusion coefficient of κ-casein in dense phase (Figure 6B).

Interestingly, we have not observed phase separation in solutions of α-casein, studied at similar experimental conditions at any concentration.^22^ It is tempting to speculate that the role of κ-casein in the formation of casein micelle may be dictated by its unique association properties and tendency to form protein condensates. Future studies of different casein mixtures should clarify its role.

## Supporting Information Available

Figures S1–S7 include information on the amino acid composition of caseins, SDS–PAGE analysis, CD spectra, reversibility test, NMR pulse sequences used in this work, and the dependence of diffusion attenuation fits with the spectrum of diffusion coefficients on the number of iterations. Additionally, *R*_0_ calculation for FRET efficiency determination is provided (PDF)

## Abbreviations

CD: circular dichroism
FRET: Förster resonance energy transfer
IDP: intrinsically disordered protein
NMR: nuclear magnetic resonance
PFG: pulsed field gradient
RF: radiofrequency
STE: stimulated-echo pulse sequence
MODSTE: modified stimulated echo pulse sequence
LLPS: liquid–liquid phase separation.
