# Supplementary material for: Translational Diffusion and Self-Association of an Intrinsically Disordered Protein κ-Casein Using NMR with Ultra-High Pulsed-Field Gradient and Time-Resolved FRET
Source: J Phys Chem B. 2024 Aug 6;128(32):7781–91. doi: 10.1021/acs.jpcb.4c03625 (PMC11331516; doi:10.1021/acs.jpcb.4c03625)
Supplement: Supplementary file 1 — jp4c03625_si_001.pdf [file jp4c03625_si_001.pdf]

SUPPORTING INFORMATION

Translational Diffusion and Self-Association of an Intrinsically Disordered Protein κ-Casein Using NMR with Ultra-High Pulsed-Field Gradient and Time-Resolved FRET.

Daria L. Melnikova[†], Venkatesh V. Ranjan[‡¶], Yuri E. Nesmelov[‡], Vladimir D. Skirda[†], Irina V. Nesmelova[‡§*]

[†]Department of Physics, Kazan Federal University, Kazan 420011, Russia; [‡]Department of Physics and Optical Sciences, [¶]Department of Chemistry, and [§]School of Data Science, University of North Carolina, Charlotte, NC 28223, USA.

SEQUENCE COMPARISON OF CASEINS

Amino acid sequences of the four bovine caseins, α_{s1}, α_{s2}, β, and κ-casein:

P02668 · CASK_BOVIN Kappa-casein
QEQNQEQPIRCEKDERFFSDKIAKYIPIQYVLSRYPSYGLNYYQQKPVALINNQFLPYPYYAKPAAVRSPA
QILQWQVLSNTVPAKSCQAQPTTMARHPHPHLSFMAIPPKKNQDKTEIPTINTIASGEPTSTPTTEAVEST
VATLEDSPEVIESPPEINTVQVTSTAV

P02663 · CASA2_BOVIN Alpha-S2-casein
KNTMEHVSSSEESIISQETYKQEKNMAINPSKENLCSTFCKEVVRNANEEEYSIGSSSEESAEVATEEVKIT
VDDKHYQKALNEINQFYQKFPQYLQYLYQGPIVLNPWDQVKRNAVPITPTLNREQLSTSEENSKKTVDM
ESTEVFTKKTKLTEEEKNRLNFLKKISQRYQKFALPQYLKTVYQHQKAMKPWIQPKTKVIPYVRYL

P02662 · CASA1_BOVIN Alpha-S1-casein
RPKHPIKHQGLPQEVLNENLLRFFVAPFPEVFGKEKVNELSKDIGSESTEDQAMEDIKQMEAESISSSEEIV
PNSVEQKHIQKEDVPSERYLGYLEQLLRLKKYKVPQLEIVPNSAEERLHSMKEGIHAQQKEPMIGVNQEL
AYFYPELFRQFYQLDAYPSGAWYYVPLGTQYTDAPSFSDIPNPIGSENSEKTTMPLW

P02666 · CASB_BOVIN Beta-casein
RELEELNVPGEIVESLSSSEESITRINKKIEKFQSEEQQQTEDELQDKIHPFAQTQSLVYPFPGPIPNSLPQNI
PPLTQTPVVVPPFLQPEVMGVSKVKEAMAPKHKEMPFPKYPVEPFTESQSLTLTDVENLHLPLPLLQSW
MHQPHQPLPPTVMFPPQSVLSLSQSKVLPVPQKAVPYPQRDMPIQAFLLYQEPVLGPVRGPFPIIV

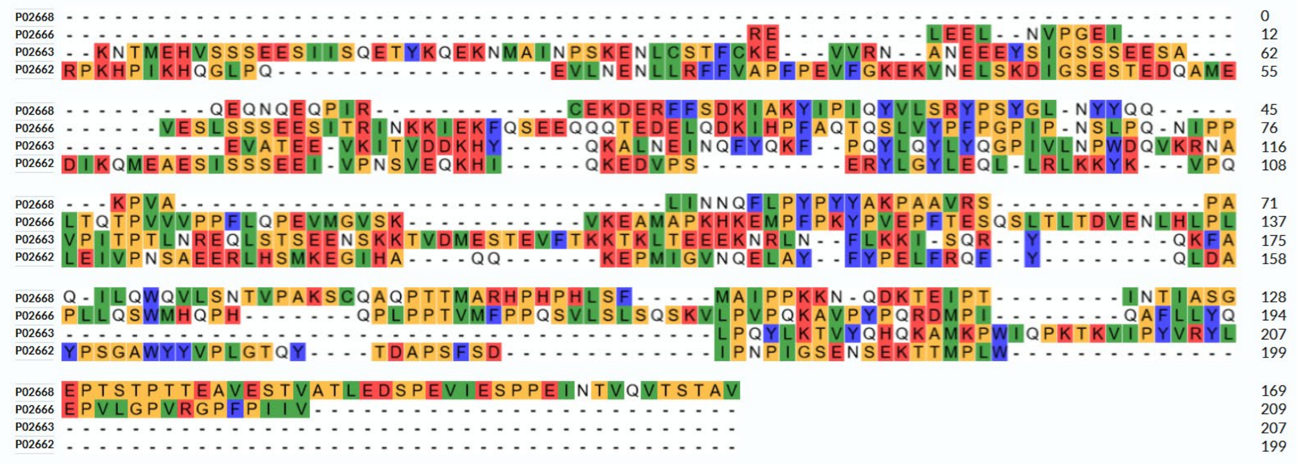

Figure S1. Amino acid sequence alignment with residues properties highlighted according to CLUSTAL color scheme: red – charged, blue – aromatic, green – aliphatic, orange – S, T, A, G, P.

GEL ELECTROPHORESIS OF κ-CASEIN

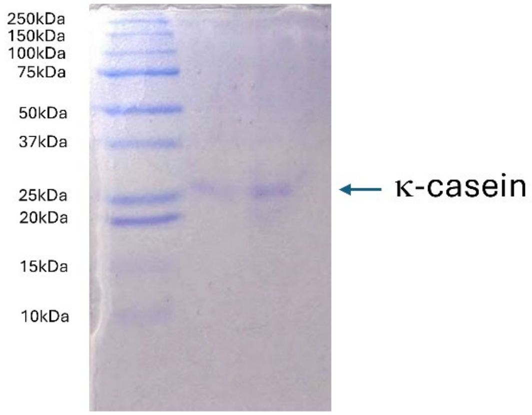

Figure S2. Denaturing SDS-PAGE analysis shows that k-casein is a pure monodisperse species.

CIRCULAR DICHROISM (CD) SPECTROSCOPY OF κ-CASEIN

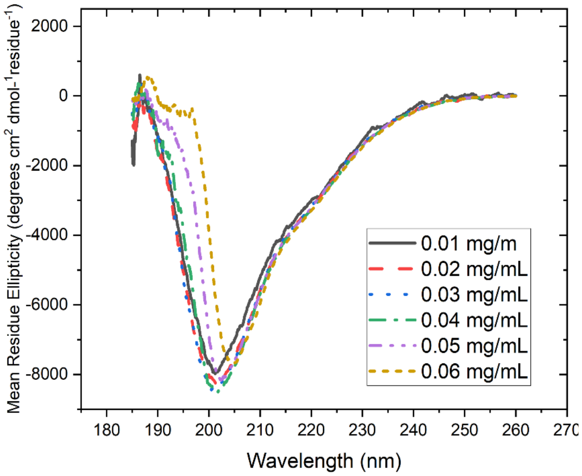

Figure S3. For the far UV CD spectroscopic measurements, the lyophilized powder of κ-casein was dissolved in H_2O at the concentrations of 0.001 to 0.006% (0.01 to 0.06 mg/mL). CD measurements were performed using a Jasco-1500 spectropolarimeter, equipped with a Peltier temperature control system. CD spectra were recorded using a 50 nm/min scan rate, a 4 s D.I.T. response, and a 1 nm bandwidth. Spectra were recorded in the range of 185–260 nm using a quartz glass cell with a path length, *l*, of 1 mm. The corresponding buffer baseline was subtracted from the spectra. Reported spectra are averages of 3–5 scans and are expressed as mean-residue molar ellipticity, [*θ*], calculated according to the following formula:

$$[\theta] = \frac{M_0 \theta_\lambda}{100 \cdot C \cdot l},$$

where M_0 is the mean residue molar mass, θ_λ is the measured ellipticity in degrees, and C is the protein concentration. CD spectra demonstrate that κ-casein does not have significant secondary structure at our experimental conditions.

STIMULATED ECHO PULSE SEQUENCES USED FOR DIFFUSION MEASUREMENTS

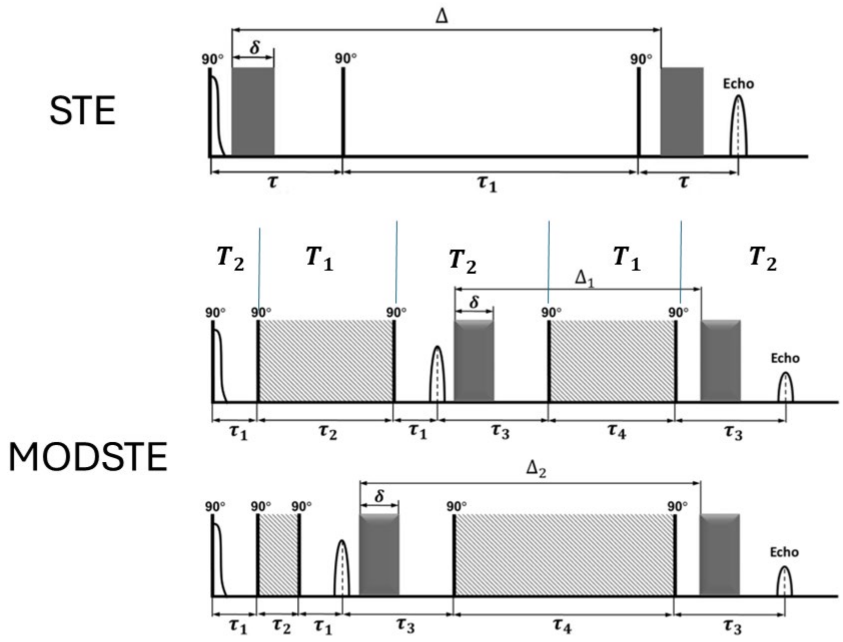

Figure S4. Stimulated echo (STE)[1] and the modified double-stimulated echo (MODSTE)[2] pulse sequences.

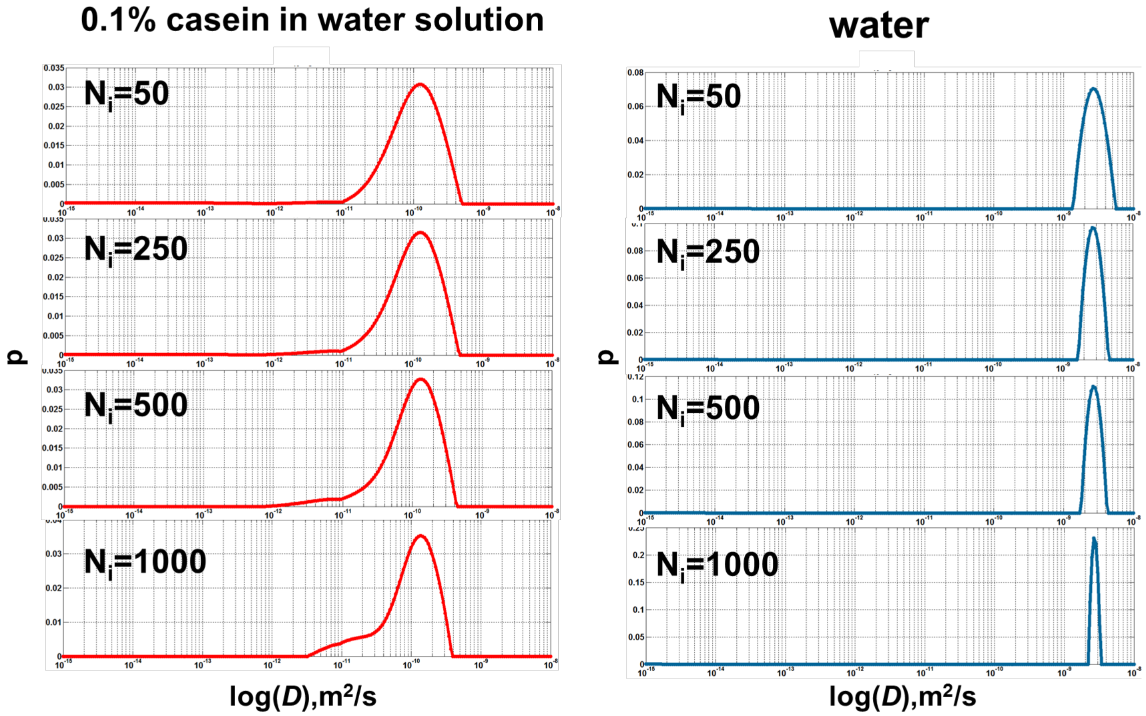

Figure S5. Spectra of diffusion coefficients of κ-casein (red, non-exponential diffusion attenuation) and water (blue, exponential diffusion attenuation) for an aqueous solution of κ-casein at a protein concentration of 0.1% as a function of the numbers of iterations N_i.

REVERSIBILITY TEST

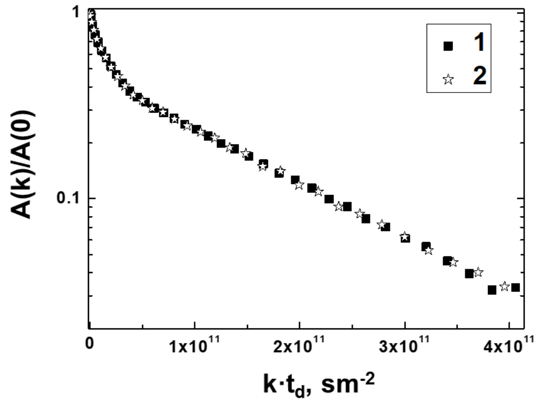

Figure S6. Diffusion attenuations recorded in κ-casein solution with protein concentration of 5%. Curve 1 corresponds to the 5 % κ-casein sample obtained by dissolving a 20 % κ-casein solution. Curve 2 corresponds to the 5 % κ-casein sample prepared directly at this concentration. $k = (\gamma\delta g)^2$, where g is the magnitude of pulsed-field gradient, $A(0)$ is the spin-echo amplitude at $g = 0$, γ is the gyromagnetic ratio for protons, δ is the gradient pulse duration and $t_d = \Delta - \delta/3$ is the diffusion time.

THE FÖRSTER DISTANCE CALCULATION

The Förster distance, $R_0 = 2.6$ nm, was calculated according to the formula[3]:

$$R_0 = 9786\left[J(\lambda)k^2\eta^4 Q_D\right]^{1/6}, \tag{1}$$

where λ is the wavelength, $J(\lambda)$ is the spectral overlap integral between the normalized donor emission spectrum $F_D(\lambda)$ and the acceptor absorption spectrum $\varepsilon_A(\lambda)$, $k^2 = 2/3$ is the probes orientation factor, $\eta = 1.4$ is the refraction index of the medium, and Q_D is the quantum yield of donor-only labeled protein ($Q_D = 0.11$). Q_D was estimated by the comparison to the quantum yield of quinine sulfate in 0.05 M H_2SO_4 at $\lambda_{ex} = 347.5$ nm (Q_S=0.51[4]), according to the equation:

$$Q_D = Q_S \frac{F_D(\lambda)/A_D(\lambda)}{F_S(\lambda)/A_S(\lambda)}, \tag{2}$$

where $F(\lambda)$ is the integral emission and $A(\lambda)$ is the absorbance at the excitation wavelength of donor-labeled protein or quinine sulfate. The analysis of time-resolved fluorescence data was performed using the software package FargoFit, designed by I.V. Negrashov, executing the global least-square fitting of multiple time-resolved luminescence waveforms using different models with ability to link fitting parameters between waveforms.

MOLECULAR DIMENSIONS OF κ-CASEIN

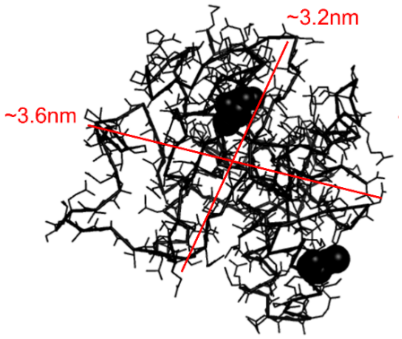

Figure S7. The linear dimensions of a κ-casein molecule were obtained using PSIPRED workbench[5], a secondary structure prediction method that incorporates two feed-forward neural networks which perform an analysis on output obtained from PSIBLAST (Position Specific Iterated – BLAST).

1. Tanner, J. E., Use of Stimulated Echo in Nmr-Diffusion Studies. *J Chem Phys* **1970,** *52* (5), 2523-&.
2. Melnikova, D. L.; Badrieva, Z. E.; Kostin, M. A.; Maller, C.; Stas, M.; Buczek, A.; Broda, M. A.; Kupka, T.; Kelterer, A. M.; Tolstoy, P. M.; Skirda, V. D., On Complex Formation between 5-Fluorouracil and β-Cyclodextrin in Solution and in the Solid State: IR Markers and Detection of Short-Lived Complexes by Diffusion NMR. *Molecules* **2020,** *25* (23).
3. Lakowicz, J. R., Principles of frequency-domain fluorescence spectroscopy and applications to cell membranes. *Subcell Biochem* **1988,** *13*, 89-126.
4. Velapoldi, R. A.; Tonnesen, H. H., Corrected emission spectra and quantum yields for a series of fluorescent compounds in the visible spectral region. *J Fluoresc* **2004,** *14* (4), 465-72.
5. Buchan, D. W. A.; Jones, D. T., The PSIPRED Protein Analysis Workbench: 20 years on. *Nucleic Acids Res* **2019,** *47* (W1), W402-W407.
